# Progress of ursolic acid on the regulation of macrophage: summary and prospect

**DOI:** 10.3389/fimmu.2025.1576771

**Published:** 2025-05-12

**Authors:** Wenjing Feng, Kehong Yang, Ying Zou, Zhaohua Xiao, Rongkang Qian, Ronghua Qian

**Affiliations:** ^1^ Key Laboratory of Vascular Biology and Translational Medicine of Hunan Province, Medical School, Hunan University of Chinese Medicine, Changsha, China; ^2^ Department of Anatomy, Anatomy Teaching Center of Hunan University of Chinese Medicine, Changsha, China; ^3^ Xiangya Hospital, Central South University, Changsha, China; ^4^ Department of Integrated Traditional Chinese and Western Medicine, Qian Rongkang Clinic, Loudi, China

**Keywords:** ursolic acid, macrophage, immunoregulation, inflammation, cytokine

## Abstract

Ursolic acid (UA), a prevalent pentacyclic triterpenoid found in numerous fruits and herbs, has garnered significant attention for its vital role in anti-inflammatory processes and immune regulation. The study of immune cells has consistently been a focal point, particularly regarding macrophages, which play crucial roles in antigen presentation, immunomodulation, the inflammatory response, and pathogen phagocytosis. This paper reveals the underlying regulatory effects of UA on the function of macrophages and the specific therapeutic effects of UA on a variety of diseases. Owing to the superior effect of UA on macrophages, different types of macrophages in different tissues have been described. Through the multifaceted regulation of macrophage function, UA may provide new ideas for the development of novel anti-inflammatory and immunomodulatory drugs. However, to facilitate its translation into actual medical means, the specific mechanism of UA in macrophages and its clinical application still need to be further studied.

## Graphical Abstract

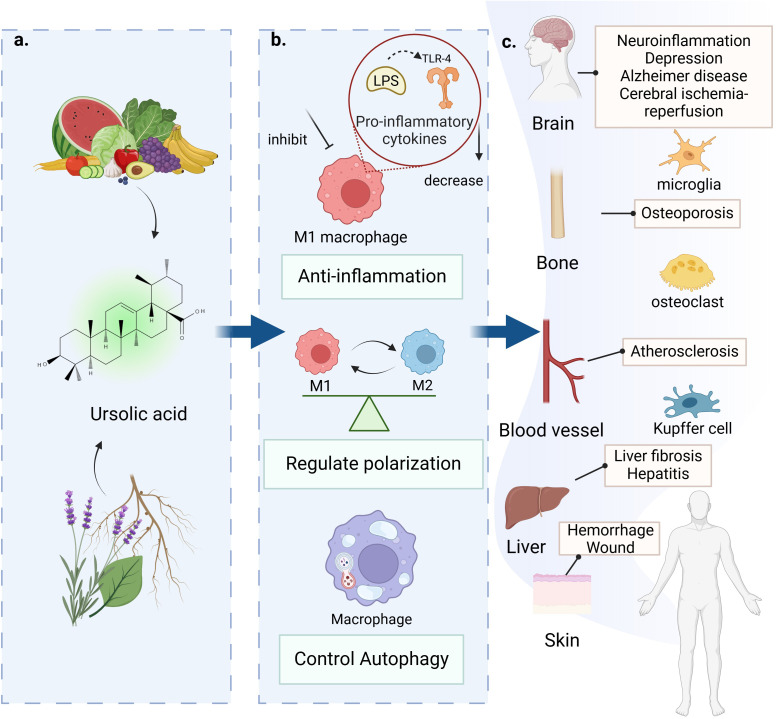

## 1 Introduction

The human immune system is a complicated defense network designed to protect the body against various pathogens, including bacteria, fungi, viruses, parasites, and aberrant cells such as cancer cells (1, 2).

Macrophages, essential cells of the innate immune system, are present in virtually every tissue throughout the human body (3). The origin of macrophages is a sophisticated process. During embryonic development, the first macrophages originate from mesenchymal progenitor cells within the yolk sac. Erythromyeloid progenitors (EMPs) subsequently colonize the fetal liver (4). They eventually differentiate into tissue specific macrophages that colonize embryonic tissue, and these cells are long-lived and self-sustaining. The production of bone marrow-derived monocytes begins after birth. Bone marrow-derived macrophages typically have a shorter lifespan and are constantly being replaced, suggesting that yolk sac derived macrophages are different from bone marrow-derived macrophages (5–7). Macrophages, which are called highly heterogeneous cell populations, exhibit different phenotypes under different stimuli and have high plasticity (8). Bacterial components such as lipopolysaccharide (LPS) and interferon-gamma (IFN-γ) polarize macrophages toward M1 macrophages, which play critical roles in acute inflammatory responses through the production of various proinflammatory cytokines that function in clearing pathogens and causing tissue damage, resulting in proinflammatory and antitumor properties. Conversely, IL-4 and IL-13 induce polarization toward M2 macrophages, which exhibit proinflammatory properties and enhance immune function (9, 10). Notably, with the advancement of omics technologies, the classification of macrophages has extended beyond two categories, revealing a more complex situation (11).

**Figure 1:**
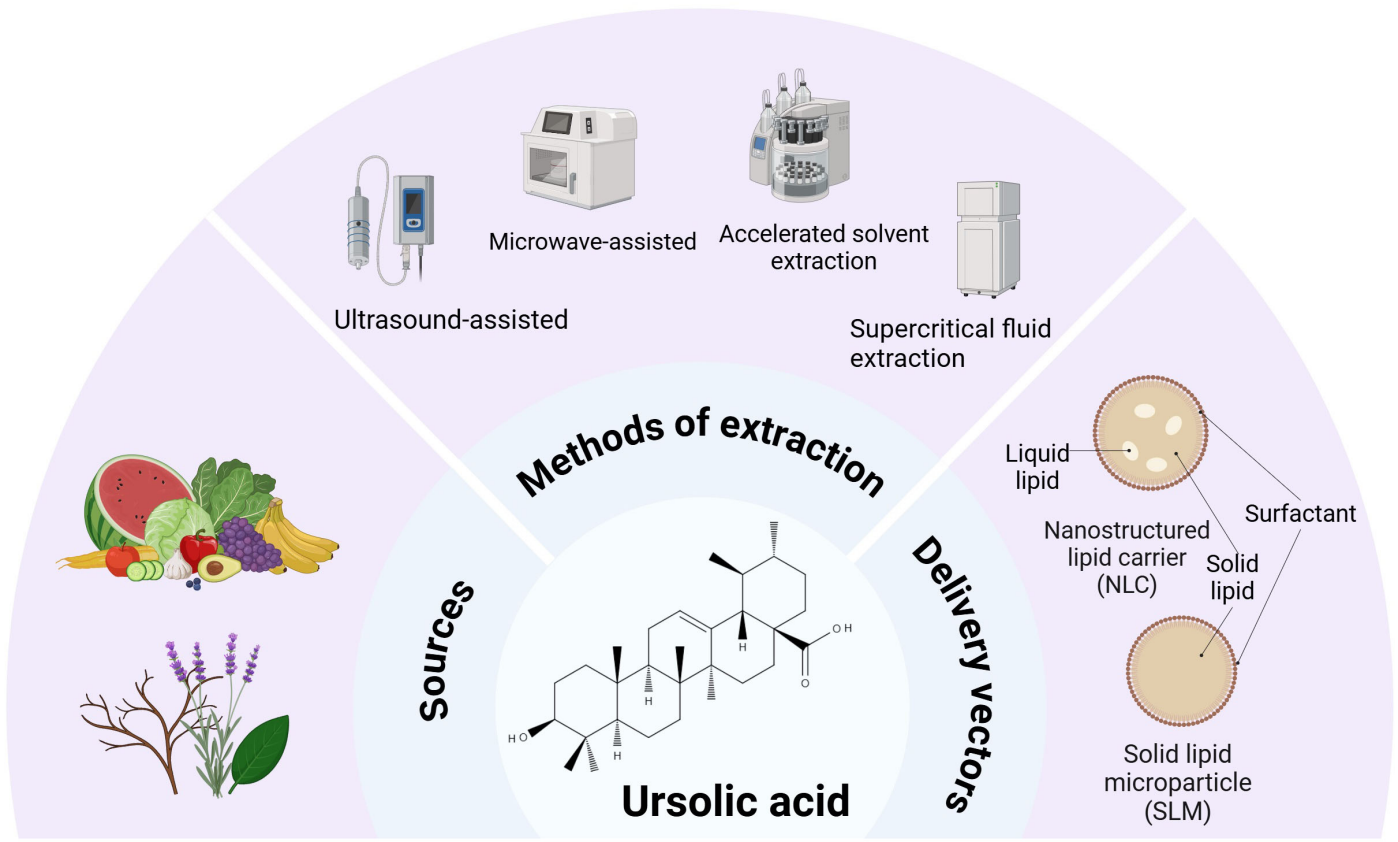
Ursolic acid sources, extraction, and delivery. UA is a pentacyclic triterpenoid compound that is widely distributed in the fruits and roots or leaves of various plants. At present, the main extraction methods for UA are ultrasonic, microwave, accelerated solvent extraction, and supercritical fluid extraction. Two common delivery methods for UA are nanostructured lipid carriers (NLCs) and solid lipid microparticles (SLMs).

Ursolic acid (UA), a natural compound, can be extracted from the stems, peels, and leaves of a variety of fruits and herbs (12) (**Figure 1**). With advancements in extraction technology, ultrasonic (13) and microwave-assisted (14) extraction of UA has gained widespread application, significantly increasing the extraction efficiency. In addition to these two techniques, methods such as accelerated solvent extraction and supercritical fluid extraction are also employed (15). The effective extraction of UA is advantageous for investigating its biological activity. UA is widely known as a pentacyclic triterpenoid compound with antitumor (16), anti-inflammatory (17), and regulatory effects on metabolic diseases, as well as cardiovascular diseases (18) but exhibits poor bioavailability. Despite the robust scientific support for UA’s pharmacological properties *in vitro* and *in vivo*, its clinical application remains constrained by inherent limitations, prompting researchers to develop multiple strategies such as solid lipid microparticles (SLMs), nanostructured lipid carriers (NLCs), and structural derivatization of UA to enhance its bioavailability. In the animal model of pulmonary tuberculosis, SLM, as a delivery carrier of UA, can increase the biological activity of UA and effectively reduce the load of Mycobacterium tuberculosis burden (Mtb) in infected alveolar macrophages (19). The water insolubility of UA limits its transport and delivery in the human body, resulting in a decreased fraction of UA available for intestinal absorption (20). The application of NLCs significantly enhances the oral bioavailability of UA, with experimental studies demonstrating that UA-loaded NLCs achieve 98.75% inhibition of parasitic infections in standardized vivo models (21). Both increased leishmanicidal activity and reduced inflammatory processes observed in the spleen and liver of animals treated with UA-NLCs can be associated with the uptake of nanoparticles by macrophages (22). Nanostructured lipid carriers loaded with *Ocimum sanctum* L. leaf extract (OLE-NLCs) were developed for improved transdermal delivery of UA for anti-arthritic therapy (23). Additionally, the anticancer potential of UA-loaded NLCs was evaluated by assessing their cytotoxic effects against the human leukemic K562 and melanoma B16 cell lines (24). Recent research on UA demonstrates that structural derivatization through the introduction of an indole ring at the C-3 position and an amide group at the C-17 position, aiming to enhance pharmacological potential, can significantly suppress LPS-induced pro-inflammatory cytokines in RAW 264.7 macrophages (25).

## 2 Effect of UA on inflammation

Macrophages serve as pivotal mediators and coordinators in the pathogenesis of chronic inflammatory disorders (26). Classically activated M1 macrophages orchestrate host defense mechanisms against bacterial, protozoan, and viral pathogens while contributing to antitumor immunity (27). These macrophages execute critical functions during acute inflammation through the release of proinflammatory mediators (28). Under homeostatic conditions, IL-10 modulates colonic macrophage activity by suppressing inflammatory responses toward gut microbiota-derived signals. This establishes IL-10 as a master anti-inflammatory cytokine. Experimental administration of UA to IL-10^-^/^-^ murine peritoneal macrophages demonstrated significant suppression of proinflammatory cytokine production (29), suggesting UA mimics IL-10-mediated immunoregulation. Furthermore, dichloromethane extracts from *Salvia connivens* leaves (DESC) exhibit anti-inflammatory properties through dual mechanisms: enhancing IL-10 biosynthesis and attenuating LPS-induced macrophage activation, with UA being identified as a principal bioactive constituent mediating these effects (30).

**Figure 2:**
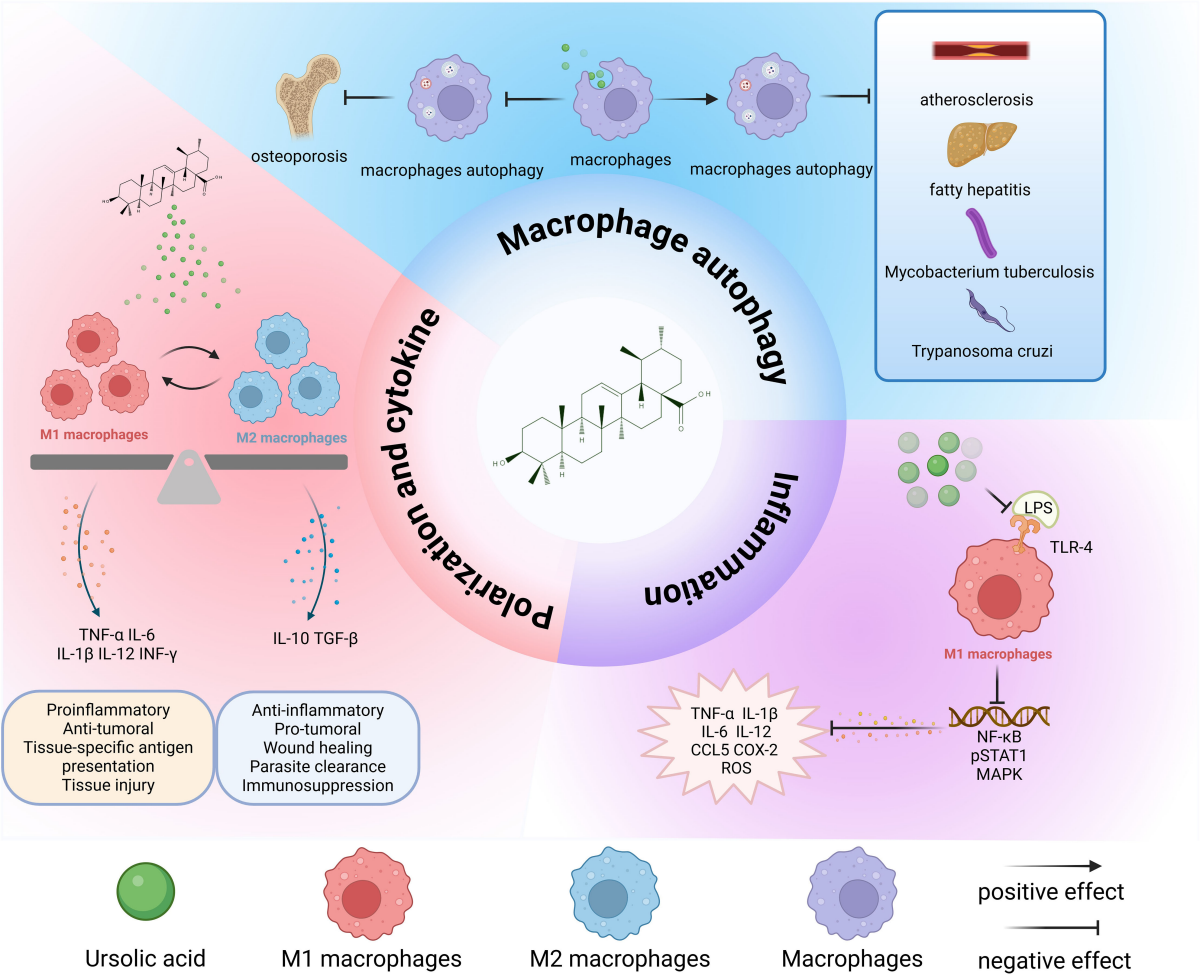
Overall effects of UA on macrophages. UA transforms M1 and M2 macrophages to each other and performs different physiological functions. Promoting macrophage autophagy is beneficial for inhibiting the pathological progression of atherosclerosis, fatty hepatitis, Mycobacterium tuberculosis infection, and parasites. The inhibition of autophagy is beneficial for the treatment of osteoporosis. UA exerts anti-inflammatory effects mainly by inhibiting the binding of LPS to TLR-4, and then inhibiting the conduction of downstream signaling pathways and reducing the release of inflammatory cytokines. TNF-α, tumor necrosis factor-α; IL-6, interleukin-6; IL-1β, interleukin-1β; IL-12, interleukin-12; IL-10, interleukin-10; LPS, lipopolysaccharide; IFN-γ, interferon-gamma; TGF-β, transforming growth factor-β; TLR4, Toll-like receptor 4; CCL5, C-C chemokine ligand 5; ROS, reactive oxygen species; COX-2, cyclooxygenase-2.

The innate immune system was originally considered to exhibit nonspecific microbial recognition; however, the identification of Toll-like receptors (TLRs) in the mid-1990s revealed that innate immunity possesses pathogen-specific recognition capabilities (31). Dysregulated TLR signaling may drive acute or chronic inflammatory responses and precipitate systemic autoimmune disorders (32). Specifically, TLR4 serves as the primary receptor for bacterial LPS. LPS-TLR4 binding activates the downstream transcription factor nuclear factor kappa B cells (NF-κB), subsequently inducing the release of proinflammatory mediators including TNF-α, IL-1β, IL-6, chemokines, proteolytic enzymes, and reactive oxygen species (33) (**Figure 2**). The activation of NF-κB plays a critical role in the release of inflammatory mediators by macrophages. Triterpenic acid extract from Eriobotrya japonica leaves (TAL), with UA as its primary component, alleviated chronic bronchitis by suppressing the nuclear translocation of the NF-κB p65 subunit in alveolar macrophages to downregulate the expression of TNF-α, interleukin-1β (IL-1β), prostaglandin E2 (PGE2), and leukotriene B4 (LTB4) (34). UA can be isolated from the seeds of Cornus officinalis and inhibits the NF-κB and MAPK signaling pathways by inhibiting the binding of TLR4 to LPS on macrophages (35). Mechanistic studies on *Sonchus oleraceus* aqueous extract revealed concurrent downregulation of TLR4 and COX-2 expression in RAW 264.7 macrophages, with UA being characterized as a principal anti-inflammatory constituent (36). 23-hydroxy-UA exhibits superior inhibitory potency against macrophage-derived NO generation, displaying concentration-dependent suppression of both COX-2 protein abundance and transcriptional output (37, 38). Psidium guajava-derived UA further attenuates intracellular ROS accumulation (39). The inflammatory cascade fundamentally involves immune cell activation and tissue infiltration, where innate immune effectors mediate tissue inflammation through phagocytic clearance or paracrine secretion of bioactive mediators (40). Macrophages uniquely orchestrate chronic inflammatory processes, particularly “metainflammation” - a metabolic inflammation continuum (41). The regulation of inflammation by UA is dynamic, likely mediated through stimulus-dependent macrophage polarization states.

## 3 Effect of UA on macrophage polarization and cytokine release

Notably, studies have demonstrated that UA participates in a dynamic regulatory process of cytokine release from macrophages, potentially associated with macrophage polarization (**Table 1**). The two primary macrophage subtypes, classically activated M1 and alternatively activated M2, represent polarized extremes within a spectrum of activation states (42). Further *in vitro* investigations revealed that M2-type macrophages can differentiate into distinct subsets: M2a, M2b, M2c, and M2d. M2a macrophages are commonly referred to as “wound healing” macrophages. M2c macrophages, along with M2b, are collectively termed “regulatory macrophages.” In contrast, M2d macrophages exhibit elevated expression of IL-10 and vascular endothelial growth factor (VEGF), both of which demonstrate immunosuppressive properties (43–45). Recent studies have demonstrated that TRIM29 serves as a critical regulator of macrophage polarization by modulating type I interferon (IFN) production (46, 47), PERK-mediated endoplasmic reticulum (ER) stress (48), inflammasome activation (49), and LPS-induced pro-inflammatory cytokine release (50). Notably, pathways such as ER stress (51) and inflammasome activation (52) are closely linked to the anti-inflammatory and immunomodulatory effects of UA. However, whether TRIM29 directly participates in UA-mediated regulation of macrophage polarization remains unclear. Future investigations should explore the interplay between UA and TRIM29-associated molecular networks to clarify its mechanistic role.

**Table 1:** Effects of different concentrations of UA at different durations on the release of cytokines from macrophages.

Test models	Type of drugs	Method of experiment	Dose	Duration	Major results	References
RAW264.7	UA	ELISA	1μM 5μM 20μM	12 h	TNF-α↑IL-6↑	(53)
RAW264.7	UA	RT–PCR	1μM 5μM 10μM	6 h	TNF-α↑	(56)
H37RV-J774A.1	UA	ELISA	0.625μM 2.5μM	3 h 24 h 48 h 72 h	TNF-α↑TGF-β↓	(54)
Peritoneal macrophages	UA	ELISA	4μM 20μM	1 h 3 h 6 h 12 h 24 h	IL-6↑IL-1β↑	(57)
Peritoneal macrophages and RAW264.7	UA	ELISA	4μM 20μM	12 h	IL-1β↑	(58)
The mice CRC model tumor tissue	LNT-UA	ELISA	5mg/kg	1 d 5 d 9 d	TNF-α↑INF-γ↑ IL-10↓	(59)
The mice pulmonary TB model lung homogenates	UA	RT–PCR	3.75mg/kg	30 d 60 d	TNF-α↑INF-γ↑	(64)
Alveolar macrophages in a rat model of chronic bronchitis	Eriobotrya japonica leaves (50%UA)	ELISA	50 mg/kg 150 mg/kg 450 mg/kg	14 d	IL-1β↓TNF-α↓	(34)
J774A.1	DESC (47.69%UA)	RT–PCR ELISA	25μM	2 h	TNF-α↓IL-6↓ IL-1β↓IL-10↑	(30)
RAW264.7	S.oleraceus aqueous (0.49%UA)	ELISA	31.3μM	24 h	TNF-α↓IL-6↓ IL-1β↓	(36)
Peritoneal macrophages	UA	ELISA	10μM 20μM	20 h	TNF-α↓IL-6↓ IL-1β↓IL-10↑	(35)
IL-10-deficient mice peritoneal macrophages	UA	ELISA	10μM 50μM	24 h	TNF-α↓IL-6↓ IL-12↓	(29)
T.gondii-Infected RAW264.7	UA	ELISA	25μg/ml 50μg/ml 100μg/ml	24 h	TNF-α↓TGF-β↓IL-6↓IL-1β↓ IL-10↑INF-β↑ IL-12↑	(63)

↑: upregulated, ↓: downregulated.

Unlike the common anti-inflammatory effects of UA, experimental studies have revealed that UA upregulates the expression of pro-inflammatory cytokines and enhances M1 macrophage activation. Using RAW 264.7 cells as a model system, researchers demonstrated that UA enhances TNF-α and IL-6 mRNA expression in liver macrophages (53). This phenomenon may be associated with UA-induced upregulation of CD36 receptor expression on macrophages (54). UA inhibits the polarization of M2 macrophages via downregulation of the Wnt pathway, thereby exerting anti-liver cancer effects (55). Moreover, UA enhances iNOS and TNF-α gene expression through NF-κB-dependent transcriptional regulation in macrophages (56). IL-1β exhibits critical involvement in the pathogenesis of inflammatory conditions. UA activates the Raf-1/MEK/ERK and MKK3/6/p38 MAPK pathways in macrophages, which promotes IL-1β gene transcription and leads to IL-1β mRNA expression for intracellular proIL-1β production (57, 58). For colorectal cancer (CRC), we developed a self-assembled nanomedicine (LNT-UA) through a simple nanoprecipitation method, consisting of natural bioactive components UA and lentinan (LNT). LNT-UA treatment significantly enhanced the secretion of antitumor-related cytokines IFN-γ and TNF-α, while concurrently suppressing the production of the immunosuppressive cytokine IL-10 (59).

Concomitantly, UA promotes M2 macrophage polarization, thereby exerting anti-inflammatory effects. A novel dressing design, designated as CS-PVA-UA, comprises electrospun nanofibers fabricated from a chitosan (CS) and polyvinyl alcohol (PVA) blend surface-functionalized with UA for diabetic wound management. Experimental data demonstrated that CS-PVA-UA nanofiber dressings significantly inhibit LPS-induced M1 macrophage polarization, effectively restore M2 phenotypic commitment, and accelerate inflammatory resolution (60). Microglia, the resident macrophages of the central nervous system, exhibit bidirectional plasticity between neurotoxic M1 and neuroprotective M2 states (61). UA confers neuroprotection through PPARγ-mediated selective polarization of microglia toward the M2 phenotype, attenuating neuroinflammatory responses (62). Furthermore, UA manifests dual immunomodulatory activity in *Toxoplasma gondii*-infected macrophages by augmenting anti-inflammatory cytokine secretion while suppressing proinflammatory mediators (63). Collectively, macrophages orchestrate immune responses via regulation of cytokine/chemokine networks. These signaling molecules mediate immune cell recruitment to inflammatory foci while dynamically modulating the activation states of macrophages. (**Figure 2**)

## 4 Effect of UA on macrophage autophagy

Autophagy critically regulates macrophage polarization dynamics (65–69). Tumor-associated macrophages (TAMs), a specialized macrophage population recruited to tumor microenvironments, predominantly exhibit M2-like phenotypes with minor M1 subpopulations, and are mechanistically implicated in facilitating tumor progression through angiogenesis promotion and metastatic niche formation (70). Paradoxically, autophagy-mediated ferroptosis was reported by Dai et al. to drive KRAS oncoprotein internalization in macrophages, concurrently enhancing M2 polarization and macrophage cell death, thereby fostering tumor immune evasion (71). Consequently, pharmacological autophagy inhibition demonstrates therapeutic potential through dual mechanisms: suppressing M2-mediated immune escape and enhancing T cell infiltrate-mediated tumor immunoediting (72). This functional dichotomy positions autophagy as a therapeutic double-edged sword (**Figure 2**). Notably, autophagy suppression may elicit contradictory effects – inducing pro-inflammatory M1 polarization while exacerbating inflammation. In inflammatory disease contexts, UA modulates macrophage plasticity by enhancing autophagic to attenuate M1 polarization and associated inflammatory cascades.

Macrophage autophagy has been demonstrated to mitigate chronic inflammation progression and organ fibrosis through attenuation of M1 polarization (73). Mechanically, UA enhances autophagy in macrophages via upregulation of autophagy related genes *Atg5* and *Atg16L1*, consequently modulating macrophage functionality and ameliorating murine atherosclerosis (74). Age-related decline in macrophage autophagic capacity may underlie the elevated incidence of senile steatohepatitis (75) and metabolic syndrome (76) in elderly populations, highlighting the therapeutic potential of autophagy-targeted interventions for obesity-related hepatic pathologies (77). In the context of Chagas disease, UA was investigated for its modulatory effects on *T. cruzi*-infected macrophages and cardiomyocytes *in vitro*. During late stage infection characterized by intracellular parasite nest formation, UA-induced autophagy activation in both macrophages and cardiac cells was shown to mitigate parasitosis-induced tissue damage (68). Macrophage autophagy has an inhibitory effect on inflammation. UA primarily mediates its anti-inflammatory activity by suppressing the TLR4/MyD88 signaling pathway, whereas pharmacological inhibition of autophagy using 3-methyladenine (3-MA) significantly abrogates this UA-mediated suppression (78). In *Mycobacterium tuberculosis* (Mtb)-induced tuberculosis (TB), UA enhances autophagy to mitigate macrophage hyperinflammatory responses, thereby improving clinical outcomes (79). UA demonstrates bidirectional regulation of macrophage autophagy. While basal autophagy activates NFATc1 and c-Fos to promote osteoporosis pathogenesis, UA suppresses c-Fos/NFATc1 induction, inhibits osteoclastogenesis, and attenuates pathological autophagy (80).

## 5 Effect of UA on tissue macrophages

### 5.1 Skin

**Figure 3:**
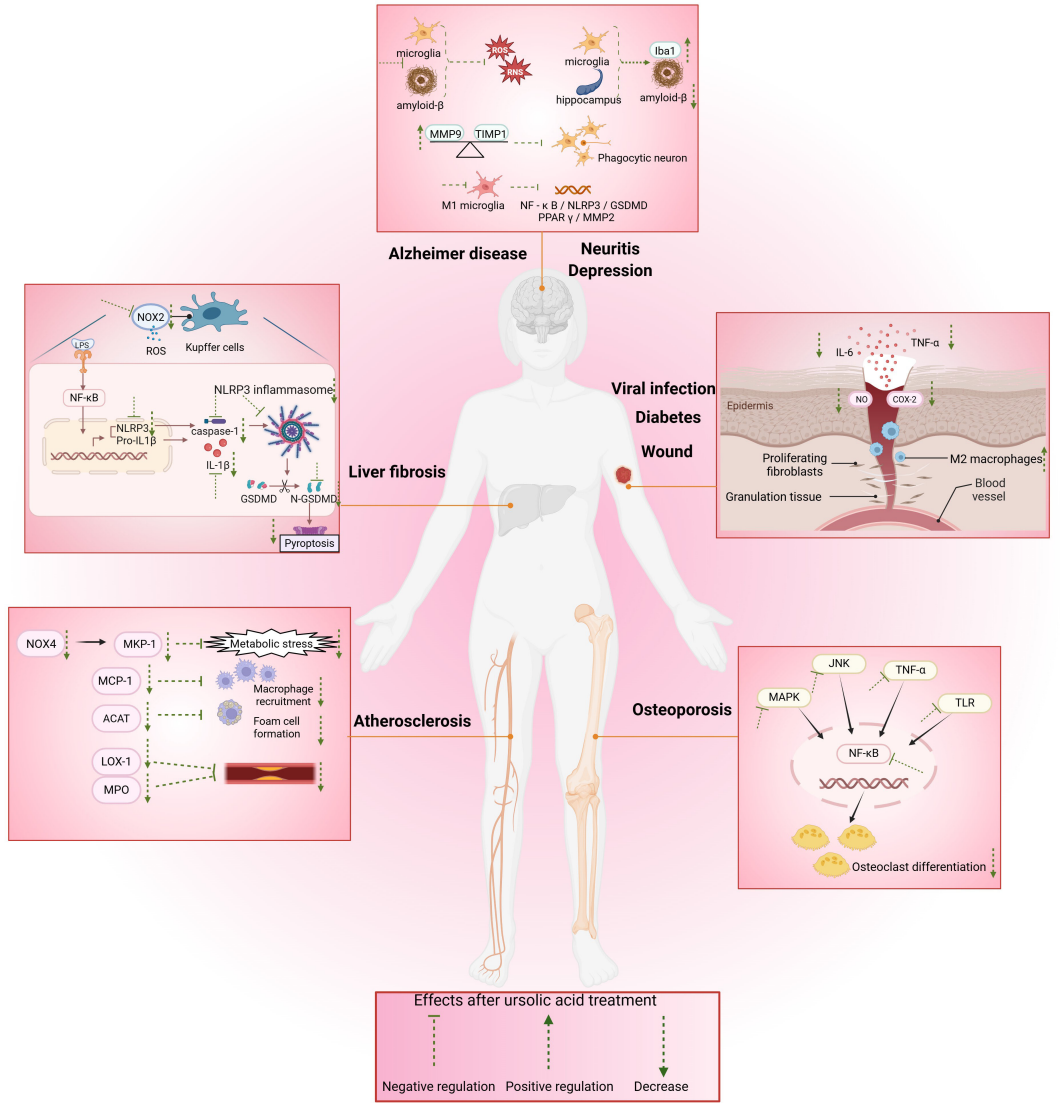
Mechanisms of the effects of UA on macrophages in various tissue diseases. UA affects macrophages, Kupffer cells, osteoclasts, and microglia to treat diabetes, parasitic infection, viral infection, liver fibrosis, atherosclerosis, osteoporosis, Alzheimer’s disease, depression, and neuritis through a variety of signaling pathways and targets. NO, nitric oxide; ROS, reactive oxygen species; COX-2, cyclooxygenase-2; TNF-α, tumor necrosis factor-α; IL-6, interleukin-6; IL-1β, interleukin-1β; NOX2, oxidase 2; NOX4, oxidase 4; NLRP3, receptors containing pyrin domain 3; GSDMD, gasdermin D; TLR4, Toll-like receptor 4; LPS, lipopolysaccharide; MPK-1, MAPK phosphatases-1; MCP-1, monocyte chemoattractant protein-1; ACAT, Acyl-coenzyme A: cholesterol acyltransferase; LOX-1, lectin-like oxidized LDL receptor-1; MPO, myeloperoxidase; Iba1, ionized calcium binding adapter molecule 1; MMP9, matrix metalloproteinases 9; PPARγ, peroxisome proliferator-activated receptor gamma; TIMP1, tissue inhibitor matrix metalloproteinase 1; MMP2, matrix metalloproteinases 2.

Hemostasis, inflammation, proliferation, and remodeling are four sophisticated and finely orchestrated physiological stages of wound healing (81). The inflammatory response is thought to involve wound healing in the first phase (82). Macrophages play pivotal roles in inflammation and wound healing by releasing cytokines such as epidermal growth factor and tumor growth factor-β(TGF-β), stimulating the proliferation of fibroblasts and keratinocytes (83), and simultaneously, they can suppress the release of inflammatory cells, facilitate the infiltration of M2 macrophages, and exert an anti-inflammatory effect (**Figure 3**).

**Figure 4:**
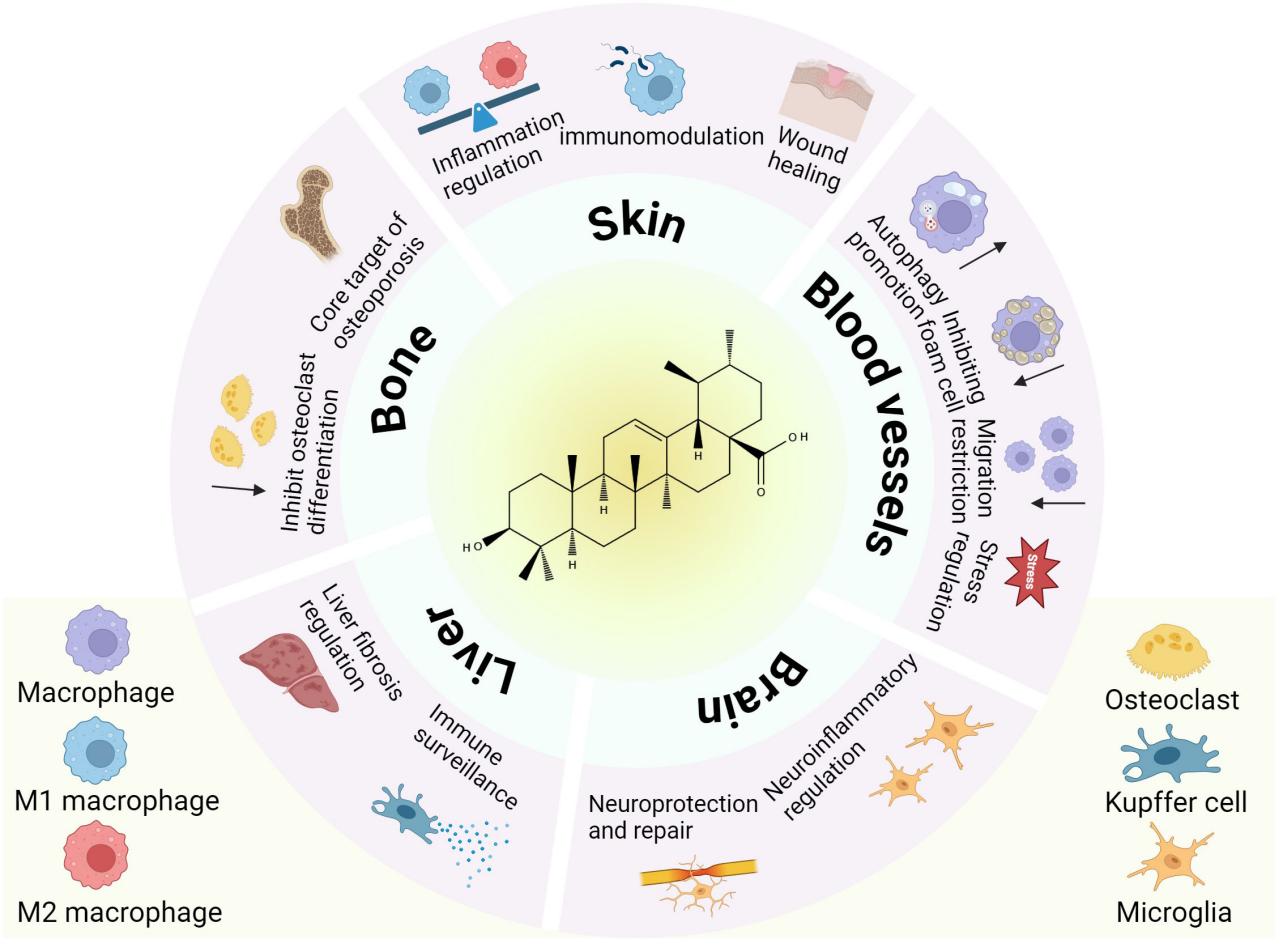
The role of tissue macrophages in disease under the influence of UA. This schematic illustrates the pleiotropic effects of UA on macrophage subtypes across diverse tissues (skin, bone, liver, brain, and blood vessels).

Researchers have used a zebrafish model to xenograft the human lung epithelial cell line A549 to study the protective effect of herb-based drug named *Coronil* against SARS-CoV-2 infection. One of the active ingredients of *Coronil* is UA, which can effectively prevent bleeding in the pelvic, dorsal, and other parts of the fish and increase the number of neutrophils and macrophages in the swim bladder to normal levels (84). *Castanea mollissima shell* (C. mollissima shell) is a traditional Chinese medicine used for wound healing and anti-inflammatory purposes. The first compound of the ethanol extracts of C. mollissima shell was identified as UA, which can reduce the number of macrophages induced by LPS, has a basic anti-inflammatory effect, and promotes wound healing (85). A new type of wound therapy for diabetes, namely, CS-PVA-UA dressings, has good shape similarity to the natural extracellular matrix (ECM) of skin collagen fibers. This type of nanofiber can promote cell adhesion and accelerate wound healing and skin regeneration *in vivo (* 60). The number of M1 macrophages is greater in diabetic wounds than in normal wounds (86), instead of M2 phenotypes, which inhibits the inflammation stage to the proliferation stage and promotes the healing of wounds (87). In a diabetic wound mouse model, the CS-PVA-UA nanofiber dressing was used to investigate its impact on skin regeneration, the results indicated that it effectively promoted revascularization, reepithelialization, collagen matrix deposition and remodeling, as well as hair follicle regeneration in diabetic wounds. This approach facilitates rapid and high quality skin wound healing in diabetic mice (44). In India, the *Shorea robusta* plant was found to treat skin injuries, including wounds and burns, and UA was identified as an effective compound of *Shorea robusta* plant with strong anti-inflammatory activity (88). It has been reported that in Leishmania, UA components extracted from the leaves of *Baccharis uncinella* upregulate Th1 cytokines, such as IL-12, can induce the differentiation and activation of IFN-γ-secreting CD4+T lymphocyte subpopulations, activate infected macrophages, and clear intracellular parasites (89–91). Experiments have shown that UA inhibits the development of skin lesions and reduces skin parasitics in BALB/c infected mice (92). Although many studies have demonstrated the effects of UA on wound healing and inflammation inhibition (**Table 2**) (**Figure 4**), some questions still need answers. Through Ikeda et al.’s finding that UA enhances iNOS and TNF-α expression in macrophages, UA-induced increases in proinflammatory mediator levels play a role in promoting the development of skin tumor formation in mice (93).

**Table 2:** Research on UA in macrophages in different tissues and diseases.

Tissues	Research objects	Type of drugs	Type of experiments	Function	References
Skin	Viral infection	Coronil	*In vivo*	Reestablished the macrophage population in the swim bladder.	(84)
	Wound	Castanea mollissima shell	*In vitro* *In vivo*	Reduced the production of TNF-α, IL-6 and NO in LPS-induced macrophages.	(85)
		Shorea robusta young leaves	*In vitro* *In vivo*	Promoted the aggregation of M2- macrophages.	(88)
	Diabetes	CS-PVA-UA	*In vitro* *In vivo*	Inhibited M1 macrophage polarization.	(60)
Liver	Liver fibrosis	UA	*In vivo*	Reduced the expression of NOXs/ROS in KCs, and improved phagocytosis of KCs.	(99)
		UA	*In vitro* *In vivo*	Inhibited KCs pyroptosis and treated liver fibrosis.	(52)
	Holestatic hepatitis; Acute hepatitis; Orthotopic liver cancer	UA-induced self-assembled nanovesicles	*In vivo*	Reduced the KCs’ phagocytosis ability toward nanomedical drugs, enhanced drug bioavailability.	(96)
	Hepatic metallothionein	UA	*In vitro*	Stimulated RAW cells to release TNF-α and IL-6, and MT expression was upregulated.	(53)
Bone	Osteoporosis	Fructus ligustri Lucidi	*In vitro*	Inhibited osteoclastogenesis in RAW264.7 cells via RANKL signaling pathways.	(105)
		UA	*In vitro* *In vivo*	Against osteoporosis by inhibiting osteoclast differentiation mediated by autophagy.	(80)
		UA	*In vitro*	Inhibited osteoclast differentiation through targeting XPO5.	(91)
	Retinoic acid-induced osteoporosis	UA	*In vivo*	Increased osteoblastic activity and reduced osteoclastic activity.	(112)
	Osteolysis	UA	*In vitro* *In vivo*	Protected against wear particle-induced osteolysis by suppressing osteoclast formation and function via NF-κB- and JNK-related signaling pathways.	(109)
	Aseptic loosening of the artificial joint	UA	*In vitro*	Inhibited the Ti wear particle-induced inflammation, osteoclastogenesis, and hydroxylapatite resorption by modifying cysteine 179 of IKKβ.	(107)
Blood vessels	Atherosclerosis	Ilex kudingcha	*In vitro* *In vivo*	Inhibited acetylated LDL induced CE accumulation (foam cell formation) in macrophages.	(120)
		UA	*In vitro*	Protected THP-1 monocytes against dysfunction by suppressing metabolic stress-induced Nox4 expression.	(115)
		UA	*In vitro* *In vivo*	Inhibited LOX-1 expressed on macrophages mediated by ROS/NF-κB signaling pathways.	(122)
		Ocimum tenuiflorum	*In vitro*	Inhibited MPO enzyme activity.	(123)
		UA	*In vitro* *In vivo*	Prevented both monocytosis induced by diabetic conditions and the phenotypical shift of blood monocytes toward a pro-inflammatory subset in diabetic mice.	(117)
		23-hydroxy ursolic acid	*In vitro* *In vivo*	Protected monocytes against metabolic stress-induced priming and dysfunction.	(114)
		Zizyphi Semen	*In vitro*	Inhibited the foaming of human macrophages.	(119)
Brain	Neuroinflammation	UA	*In vitro* *In vivo*	Inhibited microglial pyroptosis via the NF-κB/NLRP3/GSDMD pathway to alleviate neuroinflammatory.	(133)
		UA	*In vitro*	Activated PPARγ and selectively modulates microglial polarization and suppresses MMP2 formation.	(62)
	Depression	Cynomorium songaricum Rupr	*In vivo*	Inhibited M1 microglial cell polarization, and alleviated depression through the regulation of the NF-κB-NLRP3 inflammation pathway.	(129)
	Alzheimer disease	UA	*In vitro*	Blocked binding of Aβ to microglia and subsequent ROS production.	(128)
		Pyrola incarnata	*In vivo*	Improved spatial memory performance and ameliorated Aβ25–35 accumulation by activating microglia cells and upregulating Iba1 level in the hippocampus.	(130)
	Cerebral ischemia reperfusion	UA	*In vivo*	Inhibited microglia-induced neuronal cell death in an OGDR model of ischemic reperfusion injury by stabilizing the MMP9/TIMP1 imbalance.	(131)
	D-Galactose-Induced Inflammatory Response in Mouse Prefrontal Cortex	UA	*In vivo*	Reduced the number of activated microglia cells and astrocytes, decreased the expression of CD11b and glial fibrillary acidic protein, downregulated the expression of iNOS and COX-2, and decreased interleukin (IL)-1β, IL-6, and tumor necrosis factor-a levels.	(132)

### 5.2 Liver

Kupffer cells, a type of macrophage located in the hepatic venous sinuses, exhibit robust phagocytic activity(**Figure 4**). It has a strong phagocytic capacity for particulate matter, including nanoparticles (94, 95). Yuan et al. reported that liver cells are the predominant cells for the uptake of UA-induced self-assembled nanovesicles (V-UAs) and can escape the phagocytosis of Kupffer cells (96). As the early stage of cirrhosis, liver fibrosis is a complex process of fibrosis and inflammation caused by chronic liver injury (97). An imbalance between M1 and M2 macrophages has an important effect on the progression of this disease, while UA also has therapeutic effects on liver fibrosis (98). Wan et al. reported that UA alleviated liver fibrosis in mice by inhibiting the NOX2/NLRP3 inflammasome signaling pathway and thereby restraining Kupffer cell pyroptosis (52)(**Figure 3**). Another *in vitro* study demonstrated that UA can alleviate CCL4-induced liver fibrosis, meanwhile, the phagocytosis of Kupffer cells is not affected (99). Furthermore, UA has been reported to induce liver metallothionein (MT) activity (100). The underlying mechanism may be that UA acts indirectly on the liver through mediators released by Kupffer cells or the UA stimulates immune-active cells, leading to the upregulation of MT (53). Current research suggests that the mechanisms underlying the influence of UA on Kupffer cells warrant further investigation.

### 5.3 Bone

Osteoclasts and osteoblasts are important components of bone remodeling, and osteoclasts are multinucleated cells (101–103). In addition, osteoclasts are the only bone-absorbing cells in the body and play a pivotal role in the remodeling of the skeletal system (104).

UA is the main active ingredient in *Fructus Ligustri Lucidi* (FLL), an effective and well-known Chinese medicine used to treat osteoporosis. FLL ethanol extract suppresses RANKL-induced osteoclast differentiation in RAW264.7 macrophage-derived osteoclast precursors by inhibiting NF-κB signaling (105). UA mitigates LPS-induced inflammatory bone loss in mice by inhibiting RANKL-induced activation of key osteoclastogenic transcription factors, including c-Fos and NFATc1 (106). This may be one of the mechanisms by which UA inhibits osteoclast formation. And UA inhibited Ti particles induced inflammation and osteoclastogenesis by inhibiting IKKβcys-179 (107). UA was obtained via bioactivity guided fractionation of *loquat* leaves and was found to inhibit osteoclast differentiation at concentrations of 4 and 10 μg/mL (108). By inhibiting the NF-kB and JNK signaling pathways, UA decreases the number of tartrate-resistant acid phosphatase (TRAP)-positive osteoclasts (109). Through network pharmacological analysis, UA was shown to target osteoclasts mainly via a variety of signaling pathways, namely, the MAPK and tumor necrosis factor α (TNF-α) signaling pathways (110). Osteoclasts are important targets for osteoporosis (111), and osteoclast differentiation plays a key role in osteoporosis (**Figure 3**). UA may improve osteoporosis by inhibiting autophagy-mediated osteoclast differentiation (80). Additionally, UA can suppress the activity of osteoclasts while enhancing the activity of osteoblasts to facilitate bone formation, which constitutes another merit in the treatment of osteoporosis (112) (**Figure 4**). Researchers Tan et al. investigated why UA inhibits osteoclast differentiation at the molecular structure level and reported that it is likely related to C-29 and C-30 methyl groups (113).

### 5.4 Blood vessels

Macrophage autophagy exerts a protective role against early stage atherosclerosis, whereas its functional impairment in advanced disease phases exacerbates vascular inflammation, oxidative stress, and plaque necrosis, thereby accelerating disease progression (66). In the early stages of atherosclerosis, cholesterol crystals can promote the polarization of M1 macrophages and produce inflammatory responses (43).

23-Hydroxyursolic acid (23-OHUA) was identified as a potential phytochemical for the prevention and treatment of atherosclerosis. MKP-1 is a key anti-regulatory factor controlling monocyte adhesion and chemotaxis. 23-OHUA enhances MKP-1 activity in blood monocytes to a certain extent, suggesting that UA protects monocytes from metabolic priming and their transformation into a hyperchemotactic pro-atherosclerotic phenotype (114). UA exerts its anti-atherosclerotic effects by protecting blood monocytes from the effects and reprogramming induced by metabolic stress instead of lowering glucose and lipids (**Figure 3**). This situation was also mentioned in another study. UA preserves THP-1 monocyte functionality under metabolic stress through suppression of Nox4-mediated oxidative pathways (115). During lesion development, macrophages maintain a chronic inflammatory state (116). In animal experiments, UA may safeguard against the progression of atherosclerotic lesions in diabetic mice by restricting macrophage migratory capacity, and the reactivity of oxidative stress THP-1 monocytes to chemoattractant protein-1 (MCP-1) was increased; however, the surface expression of the MCP-1 receptor (CCR2) was not changed. UA can inhibit the chemotactic effect of oxidative stress on MCP-1 in a dose-dependent manner (117). The formation of macrophage-derived foam cells in atherosclerotic lesions is due to the transfer of free cholesterol to cholesterol esters (118). UA has been shown to inhibit the foam cell formation (119). Therefore, UA extracted from *Ilex kudingcha* significantly improved hyperlipidemia and atherosclerosis in APOE-deficient mice (120). LOX-1, a highly expressed transmembrane protein, is present in macrophages and is essential for the pathogenesis of atherosclerosis (121). UA decreases the mRNA and protein expression of LOX-1 (122). UA has been identified as an important component of basil. Myeloperoxidase (MPO) is an oxidase that is related to the pathogenesis of atherosclerosis. Basil extract can be used as an MPO inhibitor and as a nondrug treatment for atherosclerosis (123) (**Table 2**). Considering the pivotal role of macrophages in atherosclerotic diseases (**Figure 4**), UA has been a popular research topic, and UA has been shown to significantly reverse the abnormal activation of macrophages in atherosclerosis and to play a role in protecting blood vessels.

### 5.5 Brain

A series of neurological disorders are linked to oxidative damage and excessive inflammation, which are prevalent mechanisms by which UA affects these brain diseases (124) (**Figure 4**). UA has shown strong therapeutic potential in a variety of neurological diseases (125) and has a strong effect on microglial polarization, and the release of cytokines and inflammatory mediators (**Table 2**). The accumulation of amyloid beta (Aβ) in the brain represents a hallmark pathological characteristic of Alzheimer’s disease (AD). Aβ binds to receptor complexes (such as CD36) via microglia, triggering the release of proinflammatory cytokines and the production of neurotoxic reactive oxygen species, which in turn leads to neuronal degeneration (126). Microglia are closely related to the deposition site of Aβ in the brain, which activates microglia and produces a range of neurotoxins (127). UA reduces the ability of microglia to bind Aβ; however, it has no effect on the uptake capacity of microglia (128). Toll-like receptor 4 (TLR4) is expressed on the surface of microglia and mainly mediates the activation and inflammation of microglia induced by binding with lipopolysaccharide (LPS) (32). UA can inhibit LPS, and the combination of TLR4 with immune cells such as macrophages has been confirmed (35, 78). Perhaps through this route, effects on microglial function occur, and experiments are needed to confirm this hypothesis. Zhang et al. reported that UA, which has significant antidepressant activity, was one of the extracts of *Cynomorium songaricum Rupr* via LC-MS/MS analysis (129). Moreover, Li et al. reported that UA, a bioactive phytochemical of *wintergreen*, improves spatial memory performance by activating microglia and increasing Iba1 levels in the hippocampus. These findings suggest that UA improves cognitive performance in mice and holds promise as a natural treatment for neurodegenerative diseases (130). Furthermore, UA inhibits neuronal death in oxygen and glucose deprivation–reoxygenation (OGDR) models of microglia-induced ischemia–reperfusion injury (131). In the inflammatory response of brain tissue, UA can significantly reduce the number of activated microglia and reduce the level of inflammatory factors in the prefrontal cortex of D-galactose (D-gal)-treated mice, which can alleviate the brain inflammatory response (132). Both *in vivo* and *in vitro* studies have shown that UA can inhibit microglial polarization from the M2 phenotype to the M1 phenotype, significantly inhibit related pathways, reduce cytokine levels, and thus reduce the neuroinflammatory response induced by intracerebral hemorrhage (133). In LPS/IFN-γ activated microglia, UA modulates M1/M2 polarization through the PPARγ/MMP2 pathway, suggesting a potential mechanism underlying its neuroinflammatory regulatory effects (62)(**Figure 3**).

## 6 Discussion and prospects

This review details the effects of UA on the functions of macrophages, including polarization, cytokine release, and autophagy, as well as the role of ursolic acid-mediated macrophages in various diseases. UA is a potential drug in inflammatory diseases, and its internal mechanism has always been one of the research hotspots. Macrophages are an emerging target in immunotherapy. The combination of UA and macrophages is closely related to the immune escape of various inflammation-mediated diseases and tumors, which is worthy of further study.

In summary, UA strongly affects macrophages, but many questions need to be answered. Accurately controlling the regulatory effects of UA on cytokine release from macrophages for clinical treatment is one of the most important issues. Numerous studies on UA help develop immune cell medications that are more effective and less harmful, as well as new targets and avenues for expanding immunotherapy applications.
